# Identification and biochemical analysis of a novel *APOB* mutation that causes autosomal dominant hypercholesterolemia

**DOI:** 10.1002/mgg3.17

**Published:** 2013-06-13

**Authors:** Ellen R A Thomas, Santosh S Atanur, Penny J Norsworthy, Vesela Encheva, Ambrosius P Snijders, Laurence Game, Jana Vandrovcova, Afshan Siddiq, Mary Seed, Anne K Soutar, Timothy J Aitman

**Affiliations:** 1 MRC Clinical Sciences Centre, Imperial College LondonLondon, W12 0NN, United Kingdom; 2 BHF Centre of Research Excellence, Imperial College LondonLondon, W12 0NN, United Kingdom; 3 Department of Genomics of Common Disease, School of Public Health, Imperial College LondonLondon, W12 0NN, United Kingdom; 4 Charing Cross Hospital, Imperial College Healthcare NHS TrustLondon, W6 8RF, United Kingdom

**Keywords:** *APOB*, autosomal dominant hypercholesterolemia, exome sequencing, familial hypercholesterolemia, mass spectrometry

## Abstract

Patients with autosomal dominant hypercholesterolemia (ADH) have a high risk of developing cardiovascular disease that can be effectively treated using statin drugs. Molecular diagnosis and family cascade screening is recommended for early identification of individuals at risk, but up to 40% of families have no mutation detected in known genes. This study combined linkage analysis and exome sequencing to identify a novel variant in exon 3 of *APOB* (Arg50Trp). Mass spectrometry established that low-density lipoprotein (LDL) containing Arg50Trp APOB accumulates in the circulation of affected individuals, suggesting defective hepatic uptake. Previously reported mutations in *APOB* causing ADH have been located in exon 26. This is the first report of a mutation outside this region causing this phenotype, therefore, more extensive screening of this large and highly polymorphic gene may be necessary in ADH families. This is now feasible due to the high capacity of recently available sequencing platforms.

## Introduction

Autosomal dominant hypercholesterolemia (ADH) is a relatively common autosomal dominant condition with a prevalence of around 1 in 500 in the United Kingdom population. ADH is associated with a greatly increased risk of early onset cardiovascular disease; this risk is reduced by treatment with statin drugs (Neil et al. 2008). Clinical diagnosis in the United Kingdom is guided by the Simon Broome criteria based on cholesterol levels, presence of tendon xanthomata, family history of premature cardiovascular disease and hypercholesterolemia, and molecular testing to assign a diagnosis of definite or possible familial hypercholesterolemia (FH) (Scientific Steering Committee on behalf of the Simon Broome Register Group 1991). Most cases of ADH are caused by mutations in the *LDLR* gene, which encodes the low-density lipoprotein receptor (LDLR) protein, which binds LDL particles at the cell membrane and internalizes them for hepatic processing and excretion. Rare autosomal dominant gain-of-function mutations in *PCSK9* (proprotein convertase subtilin-kexin-type 9) can cause a severe form of ADH. A pathogenic mutation at residue 3527 in *APOB* (apolipoprotein B), the specific ligand for binding of LDL to the LDLR, has been known for over two decades to cause the autosomal dominant condition familial defective apolipoprotein-B100 (FDB), a form of ADH (Soria et al. 1989), and a second variant in this region, Arg3558Cys, has also been associated with hypercholesterolemia (Pullinger et al. 1995). In a recent study, exome sequencing identified two further variants in this region of *APOB*, each in one unrelated ADH family (Motazacker et al. 2012). In individuals with these mutations, LDL with the defective APOB has reduced affinity for the LDLR, impairing LDL clearance and causing hypercholesterolemia with a phenotype similar to that caused by *LDLR* mutations. *APOB* is a large and highly polymorphic gene, complicating identification of pathogenic variants. Screening of this gene in ADH families has therefore mainly focussed on the region of exon 26 around residue 3527.

The detection rate for pathogenic mutations in ADH varies depending on the diagnostic criteria and molecular techniques used, from 50% to 60% with stringent clinical criteria and full gene screening, to around 20–30% in standard lipid clinic populations (Taylor et al. 2010). A comprehensive study sequencing known genes in a cohort of children with suspected ADH in the Netherlands detected mutations in 95% of cases, suggesting that a minimum of 1 in 20 FH families has a causative mutation which is yet to be identified (van der Graaf et al. 2011).

Exome sequencing has become established as an effective tool for gene identification in Mendelian disease, particularly in combination with linkage analysis, as it provides a comprehensive list of candidate variants in linked genetic loci of any size, without any prior assumption of disease mechanism (Gilissen et al. 2012). We present here results of linkage and exome sequencing in a family with ADH, providing further evidence of the efficacy of this approach in this common treatable disease.

## Methods

Family members were recruited under ethics approval REC 2002/6451. Sequencing of *LDLR*, *APOB* exon 26, and *PCSK9* in I:2 identified no causative mutations. Libraries were prepared with the Agilent (Santa Clara, CA) SureSelect All Exon 50 Mb kit followed by Illumina (San Diego, CA) 100 bp paired-end sequencing (see Supplementary methods online). Read mapping used BWA (Li and Durbin 2009), and variant calling used GATK (McKenna et al. 2010). Analysis of *APOB* used the RefSeq sequence NM_000384.2. Genome-wide genotyping was carried out using Illumina Human CytoSNP-12 v2.1 beadchips; the data were analyzed with Merlin linkage analysis software (see Supplementary methods online) (Abecasis et al. 2002). LDL from three affected family members (I:2, II:2, and II:5), one unaffected family member (I:1), and an unrelated control was purified from plasma by single ultracentrifugation (Chung et al. 1980). Wild-type and mutant peptides were quantitated by mass spectrometry (see Supplementary methods online).

## Results

**Figure 1:**
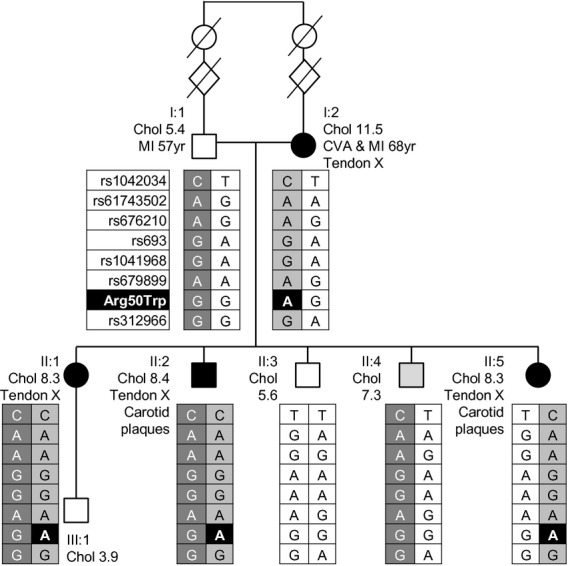
Pedigree and clinical information. Individuals with black symbols have the full phenotype of hypercholesterolemia together with tendon xanthomata; the individual with a gray symbol represents a phenocopy with high cholesterol but no xanthomata. Haplotypes within the *APOB* locus are shown for individuals with sequence data; Arg50Trp is highlighted in black in the haplotype tables. Chol, total cholesterol measurement prior to initiation of lipid-lowering treatment (mmol/L); tendon X, tendon xanthomata; MI, myocardial infarction; CVA, cerebrovascular accident.

The pedigree is shown in Figure 1, with lipid profiles given in Table 1. Linkage analysis was carried out in all individuals in generations I, II, and III shown in Figure 1, assigning affected status to the four individuals with high cholesterol and tendon xanthomata (I:2, II:1, II:2, and II:5), and unknown clinical status for II:4 with high cholesterol but no xanthomata. Two additional family members in whom DNA but no phenotypic data were available were included in the genotyping to assist with haplotype identification. Overall, 20 linkage regions were identified with maximum log of odds (LOD) score 1.2, covering a total of 245 Mb, approximately 8% of the genome.

**Table 1:** Lipid profiles of family members prior to initiation of lipid-lowering therapy

Individual	Age at measurement	BMI	Total cholesterol (mmol/L)	Triglycerides (mmol/L)	HDL (mmol/L)	LDL (mmol/L)
I1^1^	71	24	5.4			
I2	69	26	11.5	2.1	0.9	9.5
II1	50	24	8.3	0.9	1.9	6
II2	46	24	8.4	0.9	1.9	6.1
II3	57	26	5.6	1	1.3	3.8
II4	43	28	7.3	1.2	1.4	5.4
II5	49	27	8.3	1	1.9	5.9
III1	22	21	3.9	2.2	1.2	1.7

Affected individuals are shaded in gray. BMI, body mass index; HDL, high-density lipoprotein; LDL, low-density lipoprotein.

1 A full lipid profile was not available prior to lipid-lowering therapy, which was initiated for secondary prevention of coronary vascular disease in this individual some years after his myocardial infarction.

Exome sequencing generated an average of 176 million reads for each of seven family members (I:1, I:2, II:1, II:2, II:3, II:4, and II:5 in Fig. 1), of which 83% mapped within targeted regions or the 200 bp flanking these regions. Average coverage within the targeted regions was 64-fold (range 55- to 87-fold), and 86% of targeted bases were covered at minimum 10-fold. A total of 54,745 variants were identified in the seven individuals. Capillary sequencing of a subset of coding variants confirmed correct calls of 146 genotypes at 21 loci with no false positives. From the exome sequence data 4932 variants were identified within loci identified by linkage analysis, of which 1275 were nonsynonymous, essential splice site, truncating or frameshift variants. Of these, 41 segregated with the disease (i.e., were heterozygous only in the affected individuals), and 12 were novel or had minor allele frequency (MAF) less than 0.01 in dbSNP (Sayers et al. 2012), 1000 genomes project database (The 1000 Genomes Project Consortium 2010), and the NHLBI exomes project (Exome Variant Server, NHLBI Exome Sequencing Project [ESP], Seattle, WA [http://evs.gs.washington.edu/EVS/]). The 12 rare or novel segregating variants identified within linked loci are shown in Table S1 together with a summary of known functions of these genes. Eleven of the genes have known functions that are not related to lipid metabolism; the 12th was the missense variant p.Arg50Trp (c.148C>T) in *APOB* (NM_000384.2), which is a likely candidate for disease causation. Capillary sequencing confirmed the presence of this variant in the individuals with hypercholesterolemia and tendon xanthomata (I:2, II:1, II:2, and II:5) and its absence in all other available family members (I:1, II:3, II:4, and III:1). *APOB* Arg50Trp was absent from dbSNP, NHLBI ESP, and 1000 genomes project data. *In silico* tools were used to predict its effect on protein structure and function: Polyphen-2 predicted Arg50Trp to be probably damaging (score 1.0) (Adzhubei et al. 2010), the SIFT prediction was deleterious (score 0) (Kumar et al. 2009), and the Condel prediction was also of deleterious effect (score 0.762) (González-Pérez and López-Bigas 2011). This arginine residue is conserved in 27 of 29 mammals and in seven of eight of the lower species with 1:1 orthologues of this region of *APOB* identified by the Ensembl database. Overall, these *in silico* predictions suggest a functional effect of this variant. Haplotypes within the *APOB* locus are shown in Figure 1.

**Figure 2:**
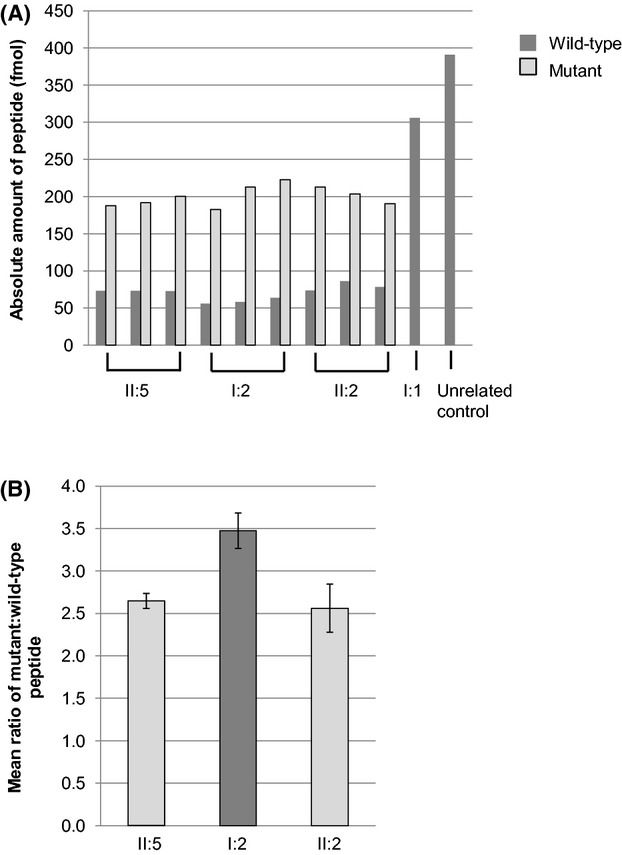
Mass spectrometry of APOB peptides. Following digestion with GluC, wild-type peptide (NVSLVCPKDATRFKHLRKYTYNYE) was present in all samples, and mutated peptide (NVSLVCPKDATRFKHLWKYTYNYE) was present only in affected individuals II:5, I:2, and II:2 and not in unaffected family members or the unrelated control. The abundance of the two peptides was quantified from the peak intensities using synthetic peptides in three replicate experiments (A). In all patient samples, the mutant peptide was present at 2.5- to 3.5-fold greater concentration than wild-type peptide (B).

*APOB* encodes the protein component of the LDL particle, forming a shell around a core of lipid molecules. LDL is cleared from the circulation by interaction with the LDLR, and this interaction has been shown to be disrupted by the FDB-causing mutations Arg3527Gln, Arg3527Trp, and Arg3558Cys (Gaffney et al. 1995; Pullinger et al. 1995). One of the methods used to study the effects of these FDB mutations is determination of the mutant:wild-type protein ratio in the circulation. Defective interaction between LDL and the LDLR results in reduced clearance of mutant LDL, while wild-type LDL is cleared normally from the circulation. Wild-type LDL therefore represents less than 50% of circulating LDL. For the Arg3527Gln mutation, the mutant:wild-type ratio has been found to be around 70:30, while for the Arg3558Cys mutation the ratio averaged 59:41 using dynamic laser light scattering (Pullinger et al. 1995). We determined the abundance of mutant and wild-type LDL in individuals with Arg50Trp and control samples using mass spectrometry (Fig. 2). The mutant peptide was present at concentrations 2.5- to 3.5-fold higher than wild-type peptide in all three affected individuals, giving a ratio of mutant:wild-type peptide between 72:28 and 78:22. The technique used to generate these ratios was different from previously reported techniques and therefore the ratios may not be directly comparable, but these results suggest that mutant APOB accumulates in the circulation as with other hypercholesterolemic *APOB* mutations, most likely due to defective hepatic uptake and clearance.

## Discussion

In recent years, exome sequencing of large numbers of individuals has revealed many thousands of coding variants in every genome, and distinguishing pathogenic variants among these has proved a significant challenge. However, combining exome sequencing with linkage data in this family reduced the number of candidate variants by 91%, and applying knowledge of the inheritance pattern of the disease allowed further reduction by focusing on segregating variants. Increasing availability of databases of rare coding variation improves detection of novel variants, although the databases may contain genuine pathogenic variants in some phenotypes, and must therefore be used with caution when excluding variants from further analysis. This is particularly likely with conditions such as hypercholesterolemia, as “control” individuals from these databases may not have had their lipid profile analyzed. Interpreting the potential functionality of variants identified by exome sequencing is frequently complex, especially where candidate variants are found within genes about which little is known. In this family, the most significant variant was within *APOB*, a gene which has been extensively studied in the context of hyperlipidemia, allowing *ex vivo* functional studies to be designed which were able to establish a difference between the wild-type and mutant proteins, lending strong support to this variant as the causative mutation in this family. A recent report of exome sequencing in ADH patients demonstrated retrospectively that individual I:2 from this family was included in another research cohort; *APOB* Arg50Trp was detected in this individual in this project, but no segregation or functional data was generated, and the mutation was presented as a variant of unknown significance (Futema et al. 2012).

Previous studies have used dynamic laser light scattering to establish the proportions of wild-type and variant APOB. This technique relies on the variant of interest being present on the same haplotype as a common polymorphism, which is detected by binding of the MB19 monoclonal antibody. For higher frequency mutations such as Arg3527Gln and Arg3558Cys this was an effective technique, but it is not applicable to rare variants in *APOB* because many families will be wild-type for the MB19 polymorphism, as with the family presented here. Mass spectrometry was therefore used for quantification of wild-type and mutant peptide in this family, to show allele imbalance for the circulating levels of APOB. This technique should also be adaptable to study LDL obtained from families with other rare *APOB* variants, in order to establish the range of mutations in *APOB* which cause hyperlipidemia. Previous studies have used mass spectrometry to quantify *APOB* in human samples (Agger et al. 2010), but this study represents the first report of the use of mass spectrometry to determine the ratios of wild-type and mutant proteins. Other studies have used trypsin to digest APOB, but trypsin cleaves at arginine residues and therefore a single peptide encompassing the Arg50Trp variant could not have been generated using the standard digestion approach. Digestion with GluC was used instead which generated two peptides from LDL of affected individuals with identical length, but differing in one amino acid (NVSLVCPKDATRFKHLRKYTYNYE and NVSLVCPKDATRFKHLWKYTYNYE).

In order to determine accurately the ratio of mutant to wild-type peptide, internal standards with known concentrations were used (ThermoFisher Scientific, Waltham, MA). The standard peptides had identical sequences to the peptides of interest, but contained a heavy labeled lysine causing a mass shift of 8 Da. The standard peptides were spiked into the digests and used to calculate the abundance of the endogenous peptides and determine the ratio of mutant to wild-type peptide. This allowed the ratios to be determined accurately and with high precision, with coefficients of variance (CVs) for the individual peptide abundances between 3% and 10% and CVs for the mutant:wild-type ratio between 6% and 12%. The small CVs indicate that using standard peptides provided satisfactory correction for technical variation resulting from the slight difference in the amino acid composition of the mutant and wild-type peptides, such as differential ionization or recovery.

Exon 3 of the *APOB* gene is 116 base pairs in length and contains little reported variation: one synonymous SNP (rs12720850) at residue 43, and two rare variants, Tyr56His (rs150496608) and Ala73Asp, both of which have been found in a single individual in the 6500 exome sequences in the NHLBI Exome Variant Server. The synonymous SNP rs12720850 is the only variant in exon 3 found in the 1000 genomes project database. This lack of nonsynonymous variation suggests an important functional role for this region of the protein. The APOB protein is structurally organized into five alternating β-sheet and α-helical domains. Mutations currently known to cause ADH are located in exon 26 in the β2 domain, which interacts directly with LDLR (Prassl and Laggner 2009). Exon 3 forms a small part of the first domain (βα1), which is a compact globular structure. This domain is predicted to direct hepatic assembly of lipoprotein molecules, as well as to affect the interaction of LDL particles with lipases and macrophage scavenger receptors. Detailed information on the specific role of exon 3 in these processes is not available, but the high level of amino acid conservation in this exon, together with the predicted damaging effect of Arg50Trp on protein function, and its absence in control datasets, suggest that this exon and in particular this nucleotide substitution are functionally important and the likely cause of hypercholesterolemia in this family. Two mechanisms could account for this. First, conformational changes in this highly conserved region of APOB could affect the interaction of LDL with LDLR and thereby increase levels of circulating LDL. Second, the mutant APOB protein could be protected from degradation prior to LDL particle assembly, stimulating the release of increased numbers of mutant very low density lipoprotein particles (VLDL) into the circulation, with the downstream effect of increasing LDL concentration. Triglyceride (and therefore VLDL) levels in the affected members of this family were within the normal range despite elevated LDL (Table 1). This suggests that reduced hepatic clearance of LDL due to defective interaction with LDLR is more likely than increased production of mutant lipoprotein particles, but further investigation of the exact mechanism in this family is beyond the scope of this article.

Haplotypes around the *APOB* locus are shown in Figure 1. The background haplotype on which the Arg50Trp mutation lies is identical in I:1 and I:2; it is possible that this has occurred due to the distant consanguinity in the family, which would indicate that the Arg50Trp mutation arose within the three generations above generation I in this pedigree. This would be consistent with rare variants of large effect being of recent origin in evolutionary terms. However, within 1000 genomes data, 17.4% of individuals of British origin (GBR) shared this background haplotype, and the presence of this haplotype in I:1 and I:2 may therefore be unrelated to the consanguinity in the family.

This study represents the first report of a mutation in *APOB* outside exon 26 causing ADH, suggesting that a wider range of APOB domains can influence the interaction between APOB and LDLR than was previously considered. In order to determine which regions of this large and highly polymorphic gene can be mutated to cause ADH, sequencing of all 29 coding exons of *APOB* will be required in large patient cohorts using the increased sequencing capacity of high-throughput platforms. Follow-up of rare and novel variants in families using the mass spectrometry protocol presented here will enable distinction of variants causing ADH from nonfunctional polymorphisms. This is likely to increase the number of families with a detectable molecular cause for ADH, enabling cascade screening in these families and potentially increasing the understanding of the interactions between the LDL particle and the LDLR.

## Supporting Information

**Data S1.** Online supplementary methods.

**Table S1.** Rare (MAF <0.01) segregating missense/frameshift variants mapping to regions of linkage.
