# Supplementary material for: Identification and biochemical analysis of a novel APOB mutation that causes autosomal dominant hypercholesterolemia
Source: Mol Genet Genomic Med. 2013 Jun 13;1(3):155–61. doi: 10.1002/mgg3.17 (PMC3865582; doi:10.1002/mgg3.17)
Supplement: Supplementary file 1 [file mgg30001-0155-SD1.docx]

**Supplementary Methods**

Genomic DNA was extracted from whole blood using a DNA extraction kit (Qiagen, Crawley, West Sussex, UK) and sheared using acoustic fragmentation (Covaris S2). Library preparation followed the SureSelect XT protocol for Illumina multiplexed sequencing version 1.2 (Agilent Technologies Inc., California, USA) using the Agilent SureSelect All Exon 50Mb kit. The Illumina HiSeq 2000 platform was used to generate 100bp paired-end sequence data. Read mapping used BWA ([Li and Durbin, 2009](#_ENREF_10)), and variant calling used GATK ([McKenna, et al., 2010](#_ENREF_11)), with variant filtering using Variant Quality Score Recalibration for single nucleotide variants and hard filtering for small indels.

Linkage analysis from Illumina HumanCytoSNP-12 v2.1 beadchip genotyping was carried out using MapThin software to thin the data to 2.0 SNPs per centimorgan (Howey, R and Cordell, H J; <http://www.staff.ncl.ac.uk/richard.howey/mapthin/>) followed by parametric linkage analysis using Merlin software ([Abecasis, et al., 2002](#_ENREF_1)). A rare dominant disease model was used, with disease allele frequency = 0.0001 and penetrances = 0.0001,1.0,1.0.

LDL from five individuals was purified from plasma by single ultracentrifugation ([Chung, et al., 1980](#_ENREF_4)). LDL samples were solubilised in 10% SDS and fractionated by 10% acrylamide SDS PAGE gel (BioRad, Hemel Hempstead, UK). The high molecular weight band of APOB was excised and digested with GluC. The resulting peptides were extracted in 1% formic acid in acetonitrile, lyophilized, resuspended in 0.1% trifluoroacetic acid (TFA), and solubilised via sonication. Heavy AQUA peptides (Thermo Fisher Scientific) containing wild-type and mutant sequences were reduced using 10 mM TCEP at 60°C for 1 hour and alkylated with 20 mM iodoacetamide for 1 hour at room temperature in the dark. 1.2 pmol of both peptides was added to each digestion reaction. Peptide separation was performed using an Ultimate 3000 RSLC nano-system (Thermo Fisher Scientific). After trap enrichment on PepMap C18 (100 μm × 2 cm, 5 μm, Thermo Fisher Scientific) peptides were eluted onto a PepMap C18 (75 μm × 50 cm, 2 μm, Thermo Fisher Scientific) with a linear gradient of 4-50% solvent B (80% acetonitrile, 0.1% formic acid) over 180 minutes with a constant flow of 225 nl/min. The HPLC system was coupled to a LTQ Orbitrap XL (Thermo Fisher Scientific) via a nanospray ion source (Proxeon Biosystems, Odense, Denmark) with a spray voltage set at 2.1 kV and the heated capillary set at 275°C. Full scan MS survey spectra (m/z 350-1500) were acquired in the Orbitrap with resolution of 60,000 after accumulation of 1,000,000 ions. The seven most intense ions were fragmented in the LTQ by collision induced dissociation (normalized collision energy 35%, activation Q 0.250 and activation time 30 ms) after accumulation of 30,000 ions. Data acquisition and quantification was performed using Xcalibur (Thermo Fisher Scientific). MASCOT version 2.3.0 (Matrix Science, London, UK) was used for peptide identification.
