# Supplementary material for: Identification and biochemical analysis of a novel APOB mutation that causes autosomal dominant hypercholesterolemia
Source: Mol Genet Genomic Med. 2013 Jun 13;1(3):155–61. doi: 10.1002/mgg3.17 (PMC3865582; doi:10.1002/mgg3.17)
Supplement: Supplementary file 2 [file mgg30001-0155-SD2.docx]

Supplementary Table: Rare (MAF <0.01) segregating missense/frameshift variants mapping to regions of linkage

| **Gene** | **RefSeq accession** | **Variant** | **Variant effect** | **Gene function (OMIM)*** |
| --- | --- | --- | --- | --- |
| *CACNA2D4* | NM_172364.4 | c.898G>A | p.Gly300Ser | Retinal calcium channel |
| *SLC25A23* | NM_024103.2 | c.1336A>T | p.Ile446Phe | Mitochondrial carrier with highest expression in brain and pancreas |
| *APOB* | NM_000384.2 | c.148C>T | p.Arg50Trp | Protein component of LDL |
| *OTOF* | NM_194248.2 | c.4141G>A | p.Glu1381Lys | Cochlear function |
| *GCKR* | NM_001486.3 | c.41C>T | p.Pro14Leu | Glucokinase regulatory protein regulating blood glucose |
| *SLC4A1AP* | NM_018158.2 | c.2251A>G | p.Lys751Glu | Kidney anion exchanger adaptor protein |
| *GPRIN3* | NM_198281.2 | c.1388T>C | p.Leu463Pro | Neurite outgrowth regulator |
| *ST7* | ENST00000446490^†^ | c. 1450-1459del ACTCACCTCA | p.T484PfsX39 | Tumour suppressor gene |
| *SLC30A6* | NM_017964.3 | c.937C>G | p.Leu313Val | Zinc transporter, highest expression in liver and brain in mouse |
| *MADCAM1* | ENST00000537731^†^ | c.688C>T | p.Pro230Ser | Lymphocyte adhesion receptor |
| *SLC6A12* | NM_003044.4 | c.1481G>C | p.Trp494Ser | Betaine/GABA transporter |
| *C2orf71* | NM_001029883.2 | c.1387G>T | p.Val463Phe | Photoreceptor function |

*Gene function data are from the Online Mendelian Inheritance in Man (OMIM) database, (McKusick-Nathans Institute of Genetic Medicine, Johns Hopkins University, Baltimore, MD <http://omim.org/>).

^†^Ensembl transcript ID given as no Refseq transcript ID is available for these transcripts.
